# Breaking the barrier: oral tacrolimus plus apremilast rescues acute generalized pustular psoriasis with severe comorbidities—a case report

**DOI:** 10.3389/fimmu.2026.1801541

**Published:** 2026-06-09

**Authors:** Lei Tang, Yiman He, Zeyun Qiao, Xun Zhou, Pingsheng Hao

**Affiliations:** 1 Chengdu University of Traditional Chinese Medicine, Chengdu, China; 2 Department of Dermatology and Cosmetology, Chongqing Traditional Chinese Medicine Hospital, Chongqing, China; 3 The Second Affiliated Hospital of Chongqing Medical University, Chongqing, China

**Keywords:** acute generalized pustular psoriasis, apremilast, case report, oral, tacrolimus, treatment

## Abstract

Generalized pustular psoriasis (GPP) is a severe disease. Treatment options for refractory cases with multiple comorbidities remain limited. This report describes a 72-year-old female patient with a 10-year history of plaque psoriasis. The patient experienced recurrent episodes of GPP over the past year, complicated by type 2 diabetes mellitus, hyperlipidemia, hypertension, stage 3 chronic kidney disease (CKD), and pulmonary interstitial fibrosis. Multiple prior therapies had failed, and several conventional medications were contraindicated due to comorbidities. Additionally, the patient refused further treatment with pesolimab. Oral tacrolimus combined with apremilast was administered, resulting in resolution of skin lesions and improvement in renal function.To our knowledge, this is among the first reported cases of successful treatment for recurrent acute GPP, especially refractory acute GPP, using oral tacrolimus in combination with apremilast. The observed therapeutic response suggests potential shared immunological pathways between acute GPP and CKD, which may be influenced by this combination therapy, contributing to improvements in both skin manifestations and renal function. This case provides a valuable reference for optimizing treatment regimens and investigating mechanisms in refractory GPP. Further studies are required to enhance therapeutic efficacy and minimize adverse effects.

## Introduction

A 72-year-old female patient presented with a 10-year history of psoriasis vulgaris (PV). One year before admission, generalized cutaneous pustules and fever developed after an upper respiratory tract infection (common cold), leading to a diagnosis of generalized pustular psoriasis (GPP). A single intravenous infusion of pesolimab (900 mg) was administered; the pustules markedly resolved on day 2 after treatment and fully resolved by day 10. Nine months previously, the patient experienced another episode of GPP (without fever) after consuming spicy food. Intravenous compound glycyrrhizin, oral total glucosides of paeony (TGP), topical fusidic acid cream, and halometasone ointment were administered, resulting in complete pustule resolution after 1 week. Five months ago, the patient developed extensive generalized pustules (without identifiable triggers), some merging into pustular lakes, accompanied by fever (39.5 °C). She was hospitalized in our department and received a single intravenous pesolimab infusion (900 mg), which partially controlled the pustules. Body temperature normalized on day 3 after treatment, and most pustules resolved. Subsequently, the patient experienced monthly mild flares of new pustules. These episodes were managed with oral total glycosides of Paeonia Lactiflora (TGP), compound glycyrrhizin, topical fusidic acid cream, and halometasone ointment. The patient had comorbidities, including type 2 diabetes mellitus (T2DM), hyperlipidemia, and hypertension. Three months previously, renal insufficiency [CKD (stage 3)] was diagnosed during a routine physical examination, and Shenyan Kangfu Tablets (a traditional Chinese medicine preparation for nephritis) were initiated.

**Figure 1:**
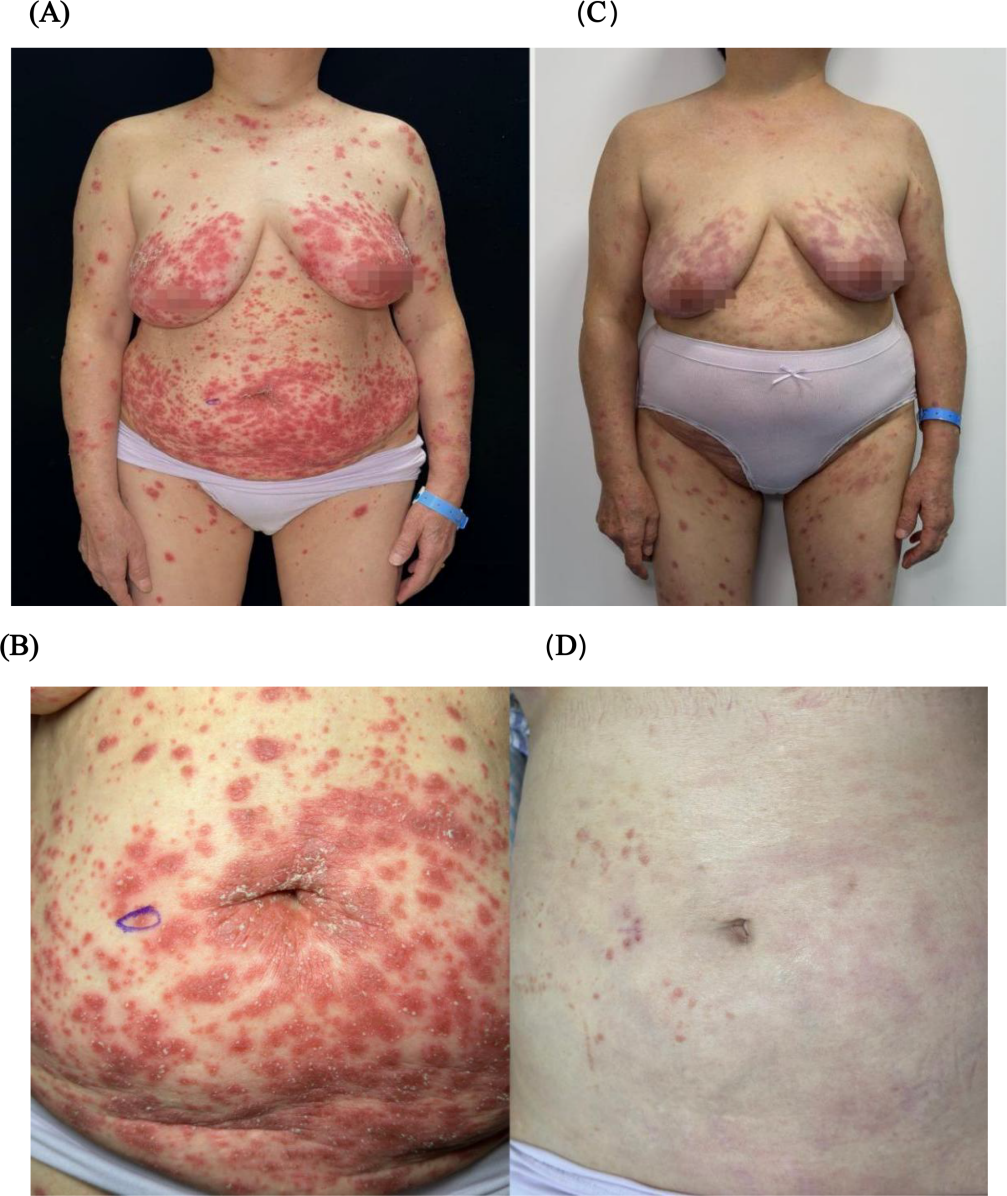
Clinical photographs showing skin lesions on the trunk and extremities before treatment **(A, B)** and on day 10 after initiation of treatment **(C, D)**.

**Figure 2:**
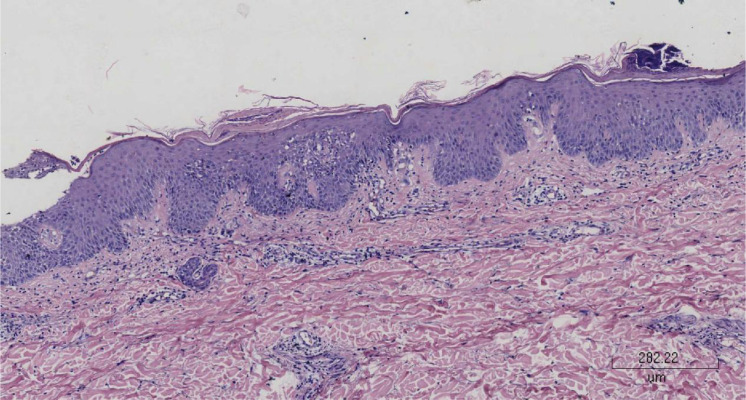
H&E staining of a pustule on the right abdomen showing epidermal hyperkeratosis, confluent parakeratosis, Kogoj spongiform pustules, neutrophilic microabscesses in the stratum corneum, and superficial perivascular lymphocytic infiltration.

**Figure 3:**
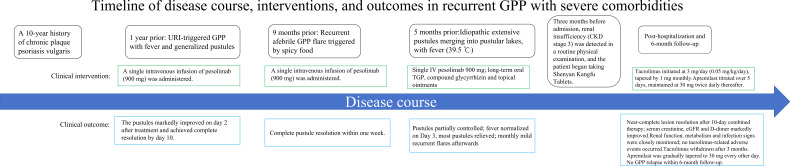
Timeline of clinical course, interventions, and outcomes in recurrent generalized pustular psoriasis (GPP) with comorbidities.

Dermatological Examination: Erythematous lesions on the extremities and trunk were accompanied by sterile pustules of varying sizes (yellow to yellowish-white). Some pustules had ruptured and dried, followed by desquamation (**Figures 1A, B**). Laboratory and Imaging Findings: Laboratory tests revealed a serum creatinine level of 195 μmol/L, an estimated glomerular filtration rate (eGFR) of 31.2 mL/min/1.73 m^2^, and a D-dimer level of 11.24 mg/L. Computed tomography (CT) imaging revealed pulmonary interstitial fibrosis. Histopathological Examination: Histopathological analysis of a pustular lesion from the right abdomen [hematoxylin-eosin (HE) staining] demonstrated epidermal hyperkeratosis with confluent parakeratosis, neutrophilic aggregates forming small abscesses in the stratum corneum, acanthosis of the spinous layer with Kogoj spongiform pustules, downward extension of rete ridges, and perivascular inflammatory infiltrates in the superficial dermis, composed predominantly of lymphocytes (**Figure 2**). Diagnosis: The patient was diagnosed with GPP, a severe psoriasis subtype associated with dysregulated innate and adaptive immunity.Treatment and Outcome: Given the patient’s renal insufficiency, pulmonary interstitial fibrosis, and hypertension, acitretin, methotrexate, and cyclosporine were excluded due to potential organ toxicity. Systemic glucocorticoids were contraindicated because of her T2DM, while topical emollients and glucocorticoids produced insufficient therapeutic effects. The patient declined retreatment with pesolimab. Considering that recurrent GPP flares were linked to inadequate control of underlying PV (a T-cell–mediated autoimmune disease), and after detailed counseling and written informed consent, combination immunomodulatory therapy was initiated.Tacrolimus was started at 3 mg/day (0.05 mg/kg/day) with a monthly reduction of 1 mg. Apremilast was introduced through a 5-day dose titration, followed by a maintenance dose of 30 mg twice daily from day 6. After 10 days of combined therapy, cutaneous lesions showed near-complete resolution (**Figures 1C, D**). Laboratory results showed improvement during treatment: serum creatinine decreased to 164 μmol/L, eGFR increased to 38.2 mL/min/1.73 m², and D-dimer declined to 1.99 mg/L. During tacrolimus treatment, serum creatinine, eGFR, electrolytes, blood pressure, blood glucose, and signs of infection were closely monitored; however, tacrolimus blood concentrations were not routinely measured due to objective limitations. No tacrolimus-related adverse events were observed. The tacrolimus induction phase lasted 3 months, after which the drug was discontinued. Three months later, apremilast was tapered to 30 mg once daily; following another 3 months of maintenance, it was further reduced to 30 mg every other day. No GPP relapse occurred during a 6-month follow-up period (**Figure 3**).

## Discussion

GPP is a severe inflammatory skin disease characterized by diffuse erythema and extensive aseptic pustules. Core features include activation of Th17 cells and abnormal activation of the cytokine axes involving IL-1, IL-17, IL-23, and IL-36 (1, 2). The mortality rate of GPP varies by population, region, and disease severity, ranging from 2% to 16% (3). GPP mortality rates are significantly higher than those of the general population and patients with plaque psoriasis. An observational study based on U.S. medical insurance data reported that GPP patients had 4.93-fold higher mortality risk than the general population and 2.31-fold higher risk than plaque psoriasis patients during a 365-day follow-up (4). During an extended follow-up (approximately 3 years), the mortality risk for GPP patients remained 3.98-fold higher than the general population and 1.49-fold higher than patients with plaque psoriasis (4). Additionally, elderly patients and those with various complications exhibit particularly high mortality rates (5, 6).

GPP manifests not only as severe skin injury but also often involves systemic inflammatory reactions leading to multi-organ damage, especially kidney injury. Studies have reported significantly increased incidences of immunoglobulin A (IgA) nephropathy and renal amyloidosis in GPP patients compared to the general population, with a 2.3-fold increased risk of progression to end-stage renal disease (ESRD) in CKD patients (7). This phenomenon is closely associated with glomerular immune-complex deposition and tubulointerstitial damage mediated by inflammatory factors (7). The immune-mediated comorbidities impose dual therapeutic demands: controlling inflammation and protecting organ function.

Current conventional GPP therapies, including methotrexate, cyclosporine, acitretin, glucocorticoids, and biologics, function primarily by modulating immune pathways (8). However, clinical use is frequently limited due to drug resistance or complications. For instance, cyclosporine increases risk by activating the renin-angiotensin system in CKD patients. While biologics target single cytokines (e.g., pesolimab targets IL-36R), existing literature (9) indicates diminished therapeutic benefits and reduced cost-effectiveness in patients with stage 3 or higher CKD. Moreover, biologics are contraindicated in certain patients with comorbid conditions such as hepatitis B and tuberculosis.

In this patient, previous pesolimab treatment provided short-term benefit but failed to control underlying psoriasis and manage multiple comorbidities effectively. Thus, exploring a combined immunomodulatory regimen that achieves rapid inflammation control in the acute phase and sustained maintenance during remission was necessary. Given these requirements, combined oral therapy with tacrolimus and apremilast was selected. This regimen was considered because it may provide complementary modulation of different immune pathways while potentially limiting organ toxicity. Tacrolimus is a widely used calcineurin (CaN) inhibitor. Its mechanism involves specifically inhibiting CaN activity, thereby blocking activated T-cell nuclear factor (NFAT) dephosphorylation pathways. This inhibits transcription and secretion of pro-inflammatory cytokines (IL-2, IFN-γ) and exerts potent immunosuppressive effects (10). Compared to cyclosporine, tacrolimus exerts weaker stimulation on juxtaglomerular organs. Previous studies have demonstrated tacrolimus’s effectiveness in controlling inflammation and reducing risks of renal toxicity and cardiovascular burden in psoriasis patients with cardiovascular disease, CKD, and metabolic syndrome (11). In this case, tacrolimus was initiated at 3 mg/day (0.05 mg/kg/day) for 4 weeks. Typical adverse events, such as tremor and hyperkalemia, did not occur. Moreover, improvements in psoriasis lesion area, GFR, serum creatinine, and D-dimer were observed, consistent with prior data in patients with stage 4 CKD-associated GPP (11).

Apremilast, a selective phosphodiesterase-4 (PDE-4) inhibitor, inhibits cyclic adenosine monophosphate (cAMP) hydrolysis, thus elevating intracellular cAMP levels and mediating anti-inflammatory and immunomodulatory effects. Specifically, apremilast treats psoriasis by: (1) inhibiting Th17 cell differentiation, thereby reducing IL-17 production, a core driver of psoriasis inflammation (12, 13); (2) decreasing pro-inflammatory cytokine release (IL-23, TNF-α), critical mediators of chronic inflammation in psoriasis (14); and (3) downregulating macrophage activation, reducing their secretion of pro-inflammatory mediators (15). Apremilast demonstrates notable efficacy in moderate-to-severe plaque psoriasis, particularly in patients responding poorly to biologics or having infectious contraindications.

Recent study has reported a case in which the patient received apremilast as maintenance therapy following inflammation control with pesolimab, suggesting its potential role in sustaining long-term immune homeostasis during the remission phase of GPP (16). Based on the literature reports (11, 16), we have fully evaluated the patient’s condition and formulated the following plan:sequential maintenance therapy with apremilast followed acute-phase tacrolimus treatment. This strategy may help reduce the risk of long-term tacrolimus-associated nephrotoxicity by limiting prolonged exposure, while sustained cAMP–PKA pathway modulation by apremilast may help maintain inflammatory control during remission. This approach offers new insights into continuous immunomodulation across acute and remission phases.

Notably, GPP has been identified as a systemic inflammatory disorder closely linked to multi-organ complications and involvement (17). Additionally, a study has emphasized abnormal IL-36 activation (core pathway in GPP), promoting renal injury by driving inflammatory responses and tissue damage. These findings provide critical clues toward understanding acute kidney injury (AKI) pathogenesis and identifying therapeutic targets (18). The temporal improvement of cutaneous lesions and renal indices following combined treatment may suggest a potential association between GPP and CKD. We hypothesize that the Th17 cell-mediated IL-17/IL-23/IL-36 axis may contribute to immune-mediated renal injury through inflammatory mechanisms and possible immune-complex deposition. In this context, tacrolimus and apremilast may have exerted complementary immunomodulatory effects on skin inflammation and renal-related inflammatory changes. However, this interpretation remains hypothetical and requires further validation in mechanistic studies and larger clinical cohorts.

In conclusion, oral tacrolimus combined with apremilast may represent a potential therapeutic option for recurrent GPP patients with multiple comorbidities, particularly when standard systemic therapies or biologics are limited. In this case, clinical improvement was observed after combined treatment, while the improvement in renal indices should be interpreted as a temporal association rather than evidence of a direct therapeutic effect. The possible immunopathological link between GPP and CKD remains hypothetical and requires further validation. Further mechanistic studies and larger clinical investigations are needed to confirm the efficacy, safety, and long-term outcomes of this regimen.

## Data Availability

The raw data supporting the conclusions of this article will be made available by the authors, without undue reservation.

## References

[1] Benezeder T Bordag N Woltsche J Falkensteiner K Graier T Schadelbauer E . Il-36-driven pustulosis: Transcriptomic signatures match between generalized pustular psoriasis (GPP) and acute generalized exanthematous pustulosis (AGEP). J Allergy Clin Immunol. (2025) 155:1913–27. doi: 10.1016/j.jaci.2025.01.046 39978684

[2] Johnston A Xing X Wolterink L Barnes DH Yin Z Reingold L . Il-1 and il-36 are dominant cytokines in generalized pustular psoriasis. J Allergy Clin Immunol. (2017) 140:109–20. doi: 10.1016/j.jaci.2016.08.056 28043870 PMC5494022

[3] Choon SE Navarini AA Pinter A . Clinical course and characteristics of generalized pustular psoriasis. Am J Clin Dermatol. (2022) 23:21–9. doi: 10.1007/s40257-021-00654-z 35061227 PMC8801409

[4] Gottlieb AB Crooke Kwiatkowski H Semeco J Lakshminarasimhan B Strober B Lebwohl M . All-cause mortality is higher in generalized pustular psoriasis (GPP) than plaque psoriasis and the general population: A US-based claims analysis. J Psoriasis Psoriatic Arthritis. (2025). doi: 10.1177/24755303251344155 40454110 PMC12125009

[5] Bellinato F Gisondi P Marzano AV Piaserico S De Simone C Damiani G . Characteristics of patients experiencing a flare of generalized pustular psoriasis: A multicenter observational study. Vaccines (Basel). (2023) 11:740. doi: 10.3390/vaccines11040740 37112652 PMC10143954

[6] Hanna ML Singer D Bender SD Valdecantos WC Wu JJ . Characteristics of hospitalizations and emergency department visits due to generalized pustular psoriasis in the United States. Curr Med Res Opin. (2021) 37:1697–703. doi: 10.1080/03007995.2021.1951192 34289766

[7] Klimko A Toma GA Ion L Mehedinti AM Andreiana I . A case report of generalized pustular psoriasis associated with IgA nephropathy. Cureus. (2020) 12:e10090. doi: 10.7759/cureus.10090 33005512 PMC7522188

[8] Lynde CW Prajapati VH Gooderham MJ Hong HC Kirchhof MG Lansang P . Considerations for treating generalized pustular psoriasis (GPP): A narrative review. Dermatol Ther (Heidelb). (2025) 15:3513–31. doi: 10.1007/s13555-025-01535-7 41066059 PMC12619870

[9] Maghfour J Elliott E Gill F Stumpf B Murina A . Effect of biologic drugs on renal function in psoriasis patients with chronic kidney disease. J Am Acad Dermatol. (2020) 82:1249–51. doi: 10.1016/j.jaad.2019.12.043 31881298 PMC7446959

[10] Maguire O Tornatore KM O'Loughlin KL Venuto RC Minderman H . Nuclear translocation of nuclear factor of activated T cells (NFAT) as a quantitative pharmacodynamic parameter for tacrolimus. Cytom A. (2013) 83:1096–104. doi: 10.1002/cyto.a.22401 24136923 PMC4018210

[11] Zhao M Huang F Tang L Zhou X Zhang M Liao M . Case report: Successful treatment of acute generalized pustular psoriasis with multiple comorbidities with oral tacrolimus. Front Immunol. (2024) 15:1354578. doi: 10.3389/fimmu.2024.1354578 38566985 PMC10985253

[12] Bianchi L Del Duca E Romanelli M Saraceno R Chimenti S Chiricozzi A . Pharmacodynamic assessment of apremilast for the treatment of moderate-to-severe plaque psoriasis. Expert Opin Drug Metab Toxicol. (2016) 12:1121–8. doi: 10.1080/17425255.2016.1206886 27376729

[13] Kumar D Sil D Kurmi BD Kumar M . Apremilast in psoriasis: Current landscape and perspectives. Recent Adv Inflammation Allergy Drug Discov. (2025). doi: 10.2174/0127722708351802250717104057 40739686

[14] De Santis M Tonutti A Isailovic N Motta F Rivara RM Ragusa R . Serum il-23 levels reflect a myeloid inflammatory signature and predict the response to apremilast in patients with psoriatic arthritis. Front Immunol. (2024) 15:1455134. doi: 10.3389/fimmu.2024.1455134 39697337 PMC11652657

[15] Subbian S Tsenova L O'Brien P Yang G Koo MS Peixoto B . Phosphodiesterase-4 inhibition alters gene expression and improves isoniazid-mediated clearance of Mycobacterium tuberculosis in rabbit lungs. PloS Pathog. (2011) 7:e1002262. doi: 10.1371/journal.ppat.1002262 21949656 PMC3174258

[16] Li Y Li Z Hu X Cao T Liu L . Combination therapy with spesolimab and apremilast for refractory generalized pustular psoriasis: a case report. Front Med (Lausanne). (2025) 12:1668675. doi: 10.3389/fmed.2025.1668675 40950991 PMC12425766

[17] Kikuchi N Kusano M Yamamoto T . Generalized pustular psoriasis as a systemic inflammatory disease: Experience with 38 Japanese cases over 15 years at a single institution. Cureus. (2025) 17:e88108. doi: 10.7759/cureus.88108 40821152 PMC12356649

[18] Nishikawa H Taniguchi Y Matsumoto T Arima N Masaki M Shimamura Y . Knockout of the interleukin-36 receptor protects against renal ischemia-reperfusion injury by reduction of proinflammatory cytokines. Kidney Int. (2018) 93:599–614. doi: 10.1016/j.kint.2017.09.017 29241623

